# Intrathecal Nicardipine as Treatment for Severe Cerebral Vasospasm After Aneurysmal Subarachnoid Hemorrhage: A Retrospective Clinical Study

**DOI:** 10.7759/cureus.71165

**Published:** 2024-10-09

**Authors:** Zachary S Smalley, Nicholas P Derrico, Paul Clark, Kenneth Winter, John H Wilkinson, Thomas R Hemphill, Hartmut Uschmann, Chad W Washington

**Affiliations:** 1 Neurosurgery, University of Mississippi Medical Center, Jackson, USA; 2 Neurosurgery, St. Vincent's Medical Center, Birmingham, USA

**Keywords:** calcium channel blocker, cerebral vasospasm, delayed cerebral ischemia, intrathecal nicardipine, sub-arachnoid hemorrhage, ventriculitis

## Abstract

Introduction

Vasospasm and delayed cerebral ischemia (DCI) are complications of aneurysmal subarachnoid hemorrhage (aSAH) and contribute up to 23% of the disability and deaths from aSAH. The use of intrathecal nicardipine (ITN) as a possible treatment for DCI has been explored with mixed results. We present a retrospective series comparing standard post-aSAH care to standard care plus ITN therapy. The primary objective of this study was to assess for any difference in functional outcome in terms of modified Rankin scale (mRS) score between the standard therapy group and ITN group at discharge and one month after discharge.

Methods

The Institutional Review Board (IRB) approval was obtained for a retrospective chart review of patients with aSAH who were treated at the University of Mississippi Medical Center between January 2012 and June 2019. The inclusion criteria included sufficient available medical documentation, aSAH with documentation of an intracranial aneurysm, and age ≥ 18. The exclusion criteria included non-aSAH, patients with insufficient medical records, and mycotic aneurysms. The decision to treat with ITN was based on the individual practice of a single neuro-intensivist in collaboration with the neurosurgical staff.

Results

A total of 385 patients were included in the study with 31 patients receiving ITN. Those within the nicardipine group presented with significantly worse Hunt and Hess grades and experienced significantly worse cerebral vasospasm, higher transcranial Doppler (TCD) velocities, higher rates of DCI, and higher rates of hydrocephalus. When controlling for placement of an external ventricular drain, the patients in the ITN group experienced higher rates of ventriculitis (10.0% vs. 2.0%, p < 0.05). There was no significant difference between the two groups in the intensive care unit (ICU) stay, hospital stay, mRS at discharge, or mRS at one-month follow-up.

Conclusion

In our series, ITN therapy did not significantly alter outcomes in terms of mRS at discharge or at one month after discharge. However, there was a significant increase in ventriculitis among patients who received this therapy.

## Introduction

In the United States, nontraumatic subarachnoid hemorrhage (SAH) has an estimated incidence of 30,000 new cases every year, with roughly 80% of nontraumatic SAH cases due to ruptured cerebral aneurysms [1]. Risk factors associated with cerebral aneurysm formation include hypertension, excessive alcohol use, and smoking with higher incidence rates in non-White populations [2]. Risk factors for aneurysmal rupture include female sex, Japanese or Finnish ancestry, hypertension, smoking, location and size of aneurysm, and sympathomimetic use [1].

Vasospasm and delayed cerebral ischemia (DCI) are complications of SAH and contribute significantly to morbidity by way of worsened neurological outcomes. Up to 23% of the disability and deaths from SAH are the result of these entities [3]. Vasospasm is radiographically present in 50%-70% of SAH cases, leading to delayed ischemic deficits in roughly one-third of patients [4]. Clinical deterioration due to DCI has been defined through consensus as focal neurological impairment or a two-point decrease in the Glasgow Coma Scale score (either on the total score or one of its components (such as eye or verbal)). This deterioration should last at least one hour, not be apparent immediately after aneurysm occlusion, and not possess known pathogenesis (i.e., not due to infection, metabolic causes, etc.) [5]. Although there are no strict criteria, diagnosis of vasospasm requires both clinical and radiographical evidence, generally the presence of vasospasm or infarction on a given radiographic modality, along with clinical deterioration or a neurological deficit [6].

Various treatment modalities have been tried to treat symptomatic vasospasm including intravascular volume manipulation, permissive and induced hypertension, mechanical angioplasty, and various intra-arterial therapies. Currently, calcium channel blockers are the most widely accepted treatment options with oral nimodipine being the only FDA-approved vasospasm treatment [3,7]. Another calcium channel blocker, nicardipine, given intrathecally has been found to have varying results. Shibuya et al. in 1994 showed no significant difference in the incidence of both symptomatic and angiographic vasospasm after intrathecal nicardipine (ITN) treatment; however, 18% of patients showed side effects such as meningitis and headaches [8]. The authors of this study did note a trend toward improvement in symptomatic vasospasm and clinical outcome. Webb et al. in 2010 showed a significant decrease in the transcranial Doppler (TCD) values following the administration of intraventricular nicardipine, which was maintained over 24 hours with continued administration [9]. In a recent large single-center study, Sadan et al. demonstrated improvement in both DCI rates (odds ratio (OR): 0.61) and favorable functional outcome (OR: 2.17 of modified Rankin scale (mRS) <= 2) [10]. Complications reported in these studies have included ventricular catheter-related infections raising concern for the association of ventriculitis with intrathecal administration of nicardipine [9-11]. This retrospective study aims to assess the effectiveness of ITN in treating cerebral vasospasm by comparing a patient population who received standard therapy alone or standard therapy plus ITN.

## Materials and methods

Study design

Institutional Review Board (IRB) approval was obtained for a retrospective chart review of patients with aneurysmal subarachnoid hemorrhage (aSAH) who were treated at the University of Mississippi Medical Center between January 2012 and June 2019. Inclusion criteria included sufficient available medical documentation, aSAH with documentation of an intracranial aneurysm on computed tomography (CT) angiography or digital subtraction angiography (DSA), and age ≥ 18. Exclusion criteria included non-aSAH (traumatic, angio-negative, arteriovenous malformation (AVM), etc.), patients with insufficient medical records, and mycotic aneurysms. The decision to administer ITN to a patient as a treatment was based on the individual practice of a single neuro-intensivist in collaboration with the neurosurgical staff. The dose (2-4 mg), frequency (1-11 doses), and duration of treatment were decided on an individual patient basis guided by practice patterns of the treating neuro-intensivist in conjunction with the attending neurosurgeon. This intervention was not administered as a prophylactic treatment but in response to worsening clinical status or as salvage therapy. Worsening clinical status included neurological decline attributable to DCI, worsening sonographic vasospasm, and in cases where other options were exhausted. Study data was collected and managed using REDCap electronic data capture tools hosted at the University of Mississippi Medical Center.

For the purposes of our study, cerebral vasospasm was defined according to TCD values, with values less than 120 cm/s considered mild, 120 cm/s-200 cm/s or Lindegaard ratio of >3 and <6 considered moderate, and over 200 cm/s or Lindegaard ratio of >6 considered severe vasospasm [6]. DCI was considered to be present when new hypodensities were seen on the CT of the head that were not related to the initial hemorrhage or when a new neurological deficit was present that had no other identifiable cause. Hydrocephalus was diagnosed with evidence of ventriculomegaly on CT head imagery along with clinical evidence of decreased consciousness or arousability and/or bradycardia not attributable to another cause. The presence of ventriculitis was confirmed with positive cerebrospinal fluid (CSF) cultures along with magnetic resonance imaging (MRI) evidence of ependymal enhancement.

Outcomes

The primary outcome of the study was mRS at discharge and one-month follow-up in aneurysmal subarachnoid patients treated with ITN in addition to standard therapy compared to those treated with standard therapy alone. Secondary analysis was performed to assess improvement in TCD values, development of cerebral vasospasm, DCI, hydrocephalus, or ventriculitis and the need for permanent CSF diversion.

Statistical analysis

The impact of ITN and baseline characteristics on clinical outcomes were evaluated with multiple statistical methods. Between group differences in the standard therapy and standard therapy + ITN groups were evaluated with Welch’s T-test. When evaluating multiple groups, analysis of variance testing was used. Univariate and multivariate regression models were used to calculate ORs of baseline patient demographics affecting clinical outcomes.

## Results

A total of 385 patients were included in the study with 31 patients in the treatment group who received ITN. The patients in the nicardipine group were significantly younger (48 vs. 54 years, p < 0.05). There was otherwise no difference with regard to gender, ethnicity, medical comorbidities, or aneurysm location (Table 1). Patients within the ITN group did present with significantly worse Hunt and Hess grades (Table 1) and experienced significantly worse cerebral vasospasm, higher TCD velocities, higher use of vasopressors, higher rates of DCI, and higher rates of hydrocephalus (Table 2).

**Table 1:** Patient demographics ITN: Intrathecal nicardipine; COPD: chronic obstructive pulmonary disease; CAD: coronary artery disease; CHF: congestive heart failure; CVD: cerebrovascular disease; Pcomm: posterior communicating artery; Acomm: anterior communicating artery; MCA: middle cerebral artery; PICA: posterior inferior cerebellar artery; ACA: anterior cerebral artery; ICA: internal carotid artery; Sup.: superior; SCA: superior cerebellar artery; PCA: posterior cerebral artery; AICA: anterior inferior cerebellar artery

	Total	Standard therapy	Standard therapy + ITN	
	(N = 385)	(N = 354)	(N = 31)	P-value
Gender				0.914
Female	264 (69%)	243 (69%)	21 (68%)	
Male	121 (31%)	111 (31%)	10 (32%)	
Age (years)	54	55	48	0.011
Ethnicity				0.937
African American	205 (53%)	188 (53%)	17 (55%)	
Asian	1 (0.3%)	1 (0.3%)	0 (0%)	
Hispanic	7 (2%)	1 (0.3%)	0 (0%)	
Native American	1 (0.3%)	1 (0.3%)	0 (0%)	
Caucasian	171 (44%)	157 (44%)	14 (45%)	
Comorbidities				
Hypertension	260 (68%)	238 (67%)	22 (71%)	0.67
Diabetes	50 (13%)	50 (14%)	0 (0%)	0.024
Smoking	184 (48%)	171 (48%)	13 (42%)	0.496
COPD	24 (6%)	23 (7%)	1 (3%)	0.47
CAD	36 (9%)	35 (10%)	1 (3%)	0.221
CHF	10 (3%)	8 (2%)	2 (6%)	0.159
CVD	37 (10%)	36 (10%)	1 (3%)	0.208
Coagulopathy	39 (10%)	35 (10%)	4 (13%)	0.593
Methamphetamine use	37 (10%)	30 (8%)	7 (23%)	0.01
Alcohol use	25 (6%)	22 (6%)	3 (10%)	0.453
Aneurysm Location				0.574
Pcomm	124 (32%)	113 (32%)	11 (35%)	
Acomm	103 (27%)	98 (28%)	5 (16%)	
MCA	51 (13%)	44 (12%)	7 (23%)	
Basilar	23 (6%)	21 (6%)	2 (6%)	
PICA	23 (6%)	22 (6%)	1 (3%)	
ACA	17 (4%)	14 (4%)	3 (10%)	
Ophthalmic	10 (3%)	10 (3%)	0 (0%)	
ICA bifurcation	7 (2%)	7 (2%)	0 (0%)	
Anterior choroidal	6 (2%)	5 (1%)	1 (3%)	
Sup. hypophyseal	6 (2%)	6 (2%)	0 (0%)	
SCA	5 (1%)	5 (1%)	0 (0%)	
PCA	4 (1%)	3 (1%)	1 (3%)	
Vertebral	4 (1%)	4 (1%)	0 (0%)	
Hunt & Hess grade				0.0014
Grade I	54 (14%)	54 (15%)	0 (0%)	
Grade II	115 (30%)	109 (31%)	6 (19%)	
Grade III	105 (27%)	97 (27%)	8 (26%)	
Grade IV	70 (18%)	62 (18%)	8 (26%)	
Grade V	41 (11%)	32 (9%)	9 (29%)	
Modified Fisher scale				0.419
Grade 0	18 (5%)	18 (5%)	0 (0%)	
Grade 1	31 (8%)	30 (9%)	1 (3%)	
Grade II	4 (1%)	4 (1%)	0 (0%)	
Grade III	163 (43%)	150 (43%)	13 (42%)	
Grade IV	167 (44%)	150 (43%)	17 (55%)	

**Table 2:** Clinical outcomes TCD: Transcranial Doppler; EVD: external ventricular drain; ICU: intensive care unit; mRS: modified Rankin scale

	Total	Standard therapy	Standard therapy + ITN	
	(N = 385)	(N = 354)	(N = 31)	P-value
Cerebral vasospasm				< 0.0001
None	178 (46%)	175 (49%)	3 (10%)	
Mild	148 (38%)	141 (40%)	7 (23%)	
Moderate/severe	59 (15%)	38 (11%)	21 (68%)	
Max TCD velocity (cm/s)	138 (58)	131 (52)	214 (67)	<0.0001
Delayed cerebral ischemia	70 (18%)	50 (14%)	20 (65%)	< 0.0001
Ventriculitis	9 (2%)	6 (2%)	3 (10%)	0.0048
(Controlling for presence of EVD)	7 (3%)	4 (2%)	3 (10%)	0.0208
Hydrocephalus	230 (60%)	199 (56%)	31 (100%)	< 0.0001
Vasopressor use	86 (22%)	66 (19%)	20 (65%)	< 0.0001
Length of stay (days)				
ICU (mean and SD)	11 ± 7	11 ± 7	14 ± 7	0.0646
Hospital (mean and SD)	17 ± 11	17 ± 11	21 ±13	0.0569
Discharge mRS				0.6757
0	4 (1%)	4 (1%)	0 (0%)	
1	53 (14%)	51 (14%)	2 (6%)	
2	69 (18%)	62 (18%)	7 (23%)	
3	99 (26%)	91 (26%)	8 (26%)	
4	77 (20%)	69 (19%)	8 (26%)	
5	47 (12%)	45 (13%)	2 (6%)	
6	36 (9%)	32 (9%)	4 (13%)	
1-month mRS				0.5435
0	25 (7%)	25 (7%)	0 (0%)	
1	93 (24%)	86 (22%)	7 (23%)	
2	82 (21%)	74 (21%)	8 (26%)	
3	56 (15%)	50 (14%)	6 (19%)	
4	47 (12%)	42 (12%)	5 (16%)	
5	37 (10%)	36 (10%)	1 (3%)	
6	43 (11%)	39 (11%)	4 (13%)	

When controlling for the placement of an external ventricular drain, the patients in the ITN group did experience higher rates of ventriculitis (10.0% vs. 2.0%, p < 0.05). Ventriculitis was found to be associated with male gender (OR: 4.5 (1.1-18.5), p = 0.0212), methamphetamine use (OR: 5.0 (1.2-21), p = 0.0145), and ITN treatment (OR: 6.2 (1.5-26.2), p = 0.0048) on univariate analysis. The development of ventriculitis was associated with ITN (OR: 6.4 (1.5-28), p = 0.0132) and male gender (OR: 4.6 (1.1-19.2). DCI was also found to be associated with the use of ITN (OR: 5.8 (2.3-15), p = 0.0002) and the use of vasopressors (OR: 12.7 (6.8-24), p < 0.0001) (Table 2). We found no significant difference in the ICU stay or hospital stay between the two groups. Most importantly, there was no significant difference between mRS score at discharge or at one-month follow-up (Table 2).

## Discussion

The use of ITN for the treatment of cerebral vasospasm after SAH is not novel, but there is a paucity of data on it. A prospective study from 1994 described 50 patients treated prophylactically with ITN after aneurysmal rupture and found no significant difference in symptomatic vasospasm or clinical outcomes when compared with a control group receiving standard therapy [8]. A case-control study from 2012 looked at 14 patients treated with ITN and found that there was a significant decrease in TCD values after treatment, but no significant difference in clinical outcome compared to the control group receiving standard therapy [12]. Sadan et al. in 2021 found that ITN decreased TCD velocities in 77.3% of treated patients, reduced the risk by 0.61 of DCI, and increased the likelihood of favorable neurologic outcome by 2.17 [10]. The available data on ITN demonstrates variable efficacy and safety in studies that are largely uncontrolled and lack standardized enrollment criteria.

Similar to several prior investigations, we found no significant difference in clinical outcomes between patients treated with ITN and those who were receiving standard vasospasm treatment. Previous studies showed improvement in TCD values within the treatment group [9-11,12-13]. Our present study found significantly increased TCD velocities in the treatment group when compared to the standard therapy group. This could reflect selection bias in the interventions used as salvage therapy. Furthermore, the patients receiving ITN had worse Hunt-Hess grades, sonographic vasospasm, hydrocephalus, and rates of DCI suggesting that they were a much sicker cohort. This is another plausible explanation for the higher rate of ventriculitis given the higher concomitant burden of critical illness.

One salient concern with any intrathecal treatment is the development of meningitis and ventriculitis. Within our study, there was a significant increase in the rate of ventriculitis in patients treated with ITN when controlling for the placement of external ventricular drains despite proper sterile precautions being taken prior to administration of the nicardipine through the drain. Additionally, in both the univariate and multivariate analysis, the development of ventriculitis was associated with ITN therapy. Multiple prior studies have reported cases of meningitis/ventriculitis in the ITN groups as well. Shibuya et. al. had 2/50 (4%) patients developed bacterial meningitis [8]. Webb et. al reported 10.9% of patients with “possible ventricular related infection” [9]. Hafeez et. al.’s 2019 systemic review reported that “infection risk appears to be in-line with studies in which rates of EVD-related infections have been reported” [11].

The development of ventriculitis as a complication of ITN creates cause for concern. The reported incidence of nosocomial ventriculitis is 0%-10.9% within the literature [8-10,12,14]. The incidence was 10% in the present study which concurs with the prior literature. The mortality of ventriculitis has been reported to be anywhere from 30% to 70%, and as a result, the risk of this serious complication needs to be weighed carefully against the benefit of treating these patients with ITN [15-16]. The study has several important limitations. It is a retrospective design, evaluating the efficacy of a primarily salvage therapy applied to patients without clearly delineated inclusion and exclusion criteria. However, the outcome of this study has changed our practice at our institution as we no longer allow the use of ITN in this patient population due to the increased risk of ventriculitis with no demonstrable clinical benefit. This article was previously posted to the Research Square Preprint Server on December 19, 2023, but publication was delayed for reanalysis in light of several positive studies [10-11,14]. After reanalysis, though there are other studies demonstrating some efficacy for this intervention in similar populations, the findings of this study may help narrow down the patient population most likely to benefit from the intervention.

## Conclusions

The use of ITN for the treatment of cerebral vasospasm after aSAH has not been found to be associated with an improvement in sonographic vasospasm or incidence of DCI in this study. Additionally, we did not find its use to be associated with improved clinical outcomes, which is consistent with most prior literature. It is associated with a significantly increased risk of ventriculitis, and therefore, this risk must be carefully considered when using this treatment option.
